# Long-term effects of sulfur mustard on civilians’ mental health 20 years after exposure (The Sardasht-Iran Cohort Study)

**DOI:** 10.1186/1477-7525-11-69

**Published:** 2013-04-24

**Authors:** Rasoul Roshan, Parvin Rahnama, Zeinab Ghazanfari, Ali Montazeri, Mohammad Reza Soroush, Mohammad Mehdi Naghizadeh, Mahdiyeh Melyani, Azadeh Tavoli, Tooba Ghazanfari

**Affiliations:** 1 Immunoregulation Research Center, Shahed University, Tehran, Iran; 2 Department of Psychology, Shahed University, Tehran, Iran; 3 Department of Midwifery, Shahed University, Tehran, Iran; 4 Psycho-Social Injuries Research Center, Ilam University of Medical Sciences, Ilam, Iran; 5 Department of Public Health, Ilam University of Medical Sciences, Ilam, Iran; 6 Mental Health Research Group, Health Metrics Research Center, Iranian Institute for Health Sciences Research, ACECR, Tehran, Iran; 7 Janbazan Medical and Engineering Research Center (JMERC), Tehran, Iran; 8 Department of Biostatistics, Fasa University of Medical Sciences, Fasa, Fars Province, Iran; 9 Department of Psychology, Faculty of Humanity Studies, Tarbiat Modares University, Tehran, Iran; 10 Department of Immunology, Shahed University, Tehran, Iran

## Abstract

**Background:**

Sulfur mustard (SM) is an alkylating agent that induces short and long term toxicity on various organs. The aim of this study was to assess the long-term psychological symptoms among samples of exposed to sulfur mustard gas compared with unexposed civilians 20 years after exposure.

**Methods:**

This historical cohort study was conducted on 495 civilians of Sardasht and Rabat in two age matched groups, including 367 sulfur mustard exposed participants from Sardasht and 128 unexposed subjects from Rabat. Psychological symptoms was assessed using the Symptom Check List-90 Revised (SCL-90-R) including measures of somatization, obsessive-compulsive, interpersonal sensitivity, depression, anxiety, hostility, phobic anxiety, paranoid ideation, and psychoticism providing three global distress indices namely: Global Severity Index (GSI), Positive Symptom Total (PST) and Positive Symptom Distress Index (PSDI). Comparison was made between exposed and unexposed civilians.

**Results:**

There were significant differences in somatization (P = 0.002), obsessive-compulsive (P = 0.031), depression (P = 0.007), anxiety (P = 0.042), and hostility (P = 0.002), between the exposed and unexposed groups. In addition there were significant differences between two groups concerning the GSI (P = 0.045) and the PSDI (P < 0.001). The differences between two groups in other subscales were not significant.

**Conclusions:**

The findings from this study showed that civilians who exposed to sulfur mustard gas were suffering from a number of psychological symptoms even 20 years after exposure. Providing mental health services and more resource allocation for this community are highly recommended.

## Background

Sulfur Mustard [bis-(2-chloroethyl) sulfide; mustard gas] is a vesicant chemical agent that can produce significant morbidity [1]. This agent was been used during the Iraq-Iran war between 1980 and 1988. Over 100,000 poorly protected soldiers suffered severe injuries as a result and roughly 45,000 soldiers continue to suffer long-lasting consequences of exposure [2]. Sardasht is a town in the north-west of Iran close to the Iraqi border. During the Iraqi war against Iran civilians of Sardasht were also exposed to mustard gas on June 28, 1987 [3].

Mustard gas can be absorbed through skin, respiratory system, genital tract, and ocular surface [2]. It affects many organs such as skin, eyes, lungs, gastrointestinal, endocrine and hematopoietic system [4]. Respiratory diseases are the major cause of long-term disability among these patients [2]. In addition, it has been also known as a DNA alkylating agent and classified as a known human carcinogen [5].

In a study on 34000 Iranians after exposure to mustard gas the most common complications were revealed in the lungs (42.5%), eyes (39.3%), and skin (24.5%) [6]. However, the effects of sulfur mustard are not restricted to these known injuries. As such it is argued that exposure to sulfur mustard gas is a traumatic event that has long-lasting effects on mental health. Mental health is a basic and integrated component of health [7]. It is well established that that there is a strong relationship between physical and mental health [8]. It is believed that the burdens of mental illnesses continue to rise worldwide and the most common mental health problems are depression and anxiety disorders [9].

Studies have reported that survivors who were exposed to both high-intensity warfare and chemical weapons were more prone to have lifetime post-traumatic stress disorder (PTSD), current PTSD, increased anxiety and depressive symptoms than those who exposed to high-intensity warfare alone [10-13]. In turn PTSD was found to be associated with increased risk of somatic symptoms and medical illnesses. Even PTSD might elevate prevalence rates of depression, anxiety and substance abuse in trauma survivors [11]. For instance, a prospective study in a non-treatment seeking sample of Iraqi war veterans revealed that the PTSD severity was associated with changes in somatic health related functioning, and with increased use of health care services [12]. In addition, it has been found that regardless of the level of physical disability, depression symptom is more evident in chemical warfare victims than those exposed to non-chemical warfare [14]. Furthermore exposure to chemical weapons has been associated with a higher prevalence of depression and anxiety disorders compared to the exposure to war-related violence alone [10].

Exposure to chemical warfare is a severe traumatic event with long-lasting effects on mental health. Survivors of chemical warfare usually suffer from psychological disorders including depression, long-term mood disorder, anxiety, post-traumatic stress disorder, and sexual dysfunction [15]. Little information exists on the topic in Iran. Thus the Sardasht-Cohort Study was initiated in order to provide help and support for civilian victims. This paper reports on psychological symptoms of civilians after twenty years exposure.

## Methods

### Study design and participants

The Sardasht-Iran Cohort Study (SICS) was designed twenty years after sulfur mustard exposure. The design and methods of the study were explained in details elsewhere [16]. In summary participants in this study were recruited from Sardasht (exposed) and Rabat (unexposed) residents. Rabat residents were exposed to intensive conventional warfare but not to chemical weapons. The exposed group was selected from the male individuals of Sardasht exposed to sulfur mustard 20 years ago based on the documents in the medical records verified by the Medical Committee of the Foundation of Martyr and Veterans Affair. In the medical records, each victim has a medical file with a numeric code. The participants were selected by systematic random sampling method from the chemical victims’ list. Since the ratio of sample size to records was 0.1, each sample was selected from every 10 records. Having studied the medical records, chemical victims were categorized into two major subgroups based on the severity of the clinical problems at the time of exposure: 1) hospitalized: victims who had moderate to severe problems and were hospitalized in Iran or sent aboard for treatment, and 2) non-hospitalized: patients who had subclinical or mild problems and were treated for acute effects on an outpatient basis. The unexposed control group was enrolled from Rabat. These participants resembled the exposed group from all aspects except exposure to sulfur mustard. Rabat is a town located 15 kilometers away from Sardasht. Both towns have similar geographical situations. They have Kurdish populations with the same religion, culture, language, and nutritional habits. The control sample included males selected by systematic random sampling from the list of latest household statistics. They were matched with the exposed group by age. After a few field trips to Sardasht and Rabat, the research team made a complete list of addresses and phone numbers of selected individuals. Civilians were then contacted by phone, and invited for interview in order to collect data on psychological symptoms. A psychiatrist interviewed all participants in a private setting in a public care center. Each interview lasted for about 30–45 minutes. Participants were included if they were 20 to 60 years old. The minimum age of 20 years was chosen because this age includes individuals who were newborns at the time of exposure. The elderly persons over 60 also were excluded to avoid unintended results due to other health conditions. In addition participants with current treatment with systemic immunosuppressive drugs, history of systemic disease before exposure based on medical records, suffering from an acute infectious disease at the time of sampling, and those who had any types of known psychological disorders such as schizophrenia and bipolar abnormalities were excluded.

### Instrument

The Symptom Check List 90 Revised (SCL-90-R) was used to collect data on psychological symptoms [17]. It includes 90 questions in 9 different psychological subscales: somatization, obsessive-compulsive, interpersonal sensitivity, depression, anxiety, hostility, phobic anxiety, paranoid ideation, and psychotic sulfur mustard. It also provides three global distress indices namely: Global Severity Index (GSI), Positive Symptom Total (PST) and Positive Symptom Distress Index (PSDI). The GSI is the average score of the 90 items of the questionnaire [18]. It is suggested to be the best single indicator of the current level of the disorder. There is a cut-off point for the GSI. An optimal cut-off point of 0.4 was considered based on the standardized test results for Iran [19]. The Positive Symptom Total (PST) is a count of all the items with non-zero responses and reveals the number of symptoms that the respondent reports experiencing. The Positive Symptom Distress Index (PSDI) is the sum of the values of the items receiving non-zero responses divided by the PST [17]. The psychometric properties of the Iranian version of SCL-90-R are well documented [20].

### Analysis

Descriptive analyses were carried out to explore the data. Statistical procedures including chi square test and t test were performed to compare exposed and unexposed civilians. Linear regression analysis was performed to examine the relationships between the scores of SCL-90-R and demographic parameters and GSI scores. A significance level equal to 0.05 or lower was adopted for all main analysis. The SPSS version 13 (SPSS Inc, Chicago IL) was used to analyze the data.

### Ethics

Ministry of Health of Iran, Janbazan Organisation, and Shahed University approved the study. Informed consent was provided from all the participants after explaining the aim of the study to them.

## Results

In all, 495 participants were studied (367 in exposed and 128 in control groups). There were no significant differences in terms of age, body mass index, marital status and employment status between the exposed and control groups (Table 1).

**Table 1:** Demographic characteristics and the SCL-90-R score of the study groups

	**Control (n = 128)**	**Exposed (n = 367)**	**P**
**Age** (Mean ± SD)	42 ± 10	44 ± 11	0.262*
**BMI** (Mean ± SD)	25.8 ± 4.0	26.3 ± 3.9	0.293*
**Marital status (number, %)**			0.892**
Single	11(8.6)	33(9.0)	
Married	117(91.4)	334(91.)	
**Education (number, %)**			<0.001**
Primary and secondary school	99(77.3)	214(58.3)	
High school and upper	29(27.3)	153(41.7)	
**Employment status (number, %)**			0.237**
Employed	73(57)	231 (62.9)	
Unemployed	55 (43.0)	136 (37.1)	
**Somatization (mean ± SD)**	1.50(0.88)	1.78(0.82)	0.002*
**Obsessive-compulsive (mean ± SD)**	1.37 (0.85)	1.55(0.79)	0.031*
**Interpersonal sensitivity (mean ± SD)**	1.08 (0.76)	1.17(0.75)	0.246*
**Depression (mean ± SD)**	1.20 (0.75)	1.42(0.81)	0.007*
**Anxiety (mean ± SD)**	1.24 (0.81)	1.41(0.80)	0.042*
**Hostility (mean ± SD)**	1.10 (0.84)	1.36(0.78)	0.002*
**Phobic anxiety (mean ± SD)**	0.76 (0.72)	0.74(0.81)	0.780*
**Paranoid ideation (mean ± SD)**	1.20 (0.80)	1.26(0.74)	0.479*
**Psychoticism (mean ± SD)**	0.78 (0.66)	0.83(0.69)	0.540*
**Global severity index (GSI) (mean ± SD)**	1.16 (0.70)	1.31(0.68)	0.045*
**Positive symptom total (PST) (Mean ± SD)**	51.10 (23.25)	52.91(19.80)	0.406*
**Positive symptom distress index (PSDI) (mean ± SD)**	1.94 (0.46)	2.14(0.48)	<0.001*

* Comparison with control group derived from t-test.

** Comparison with control group derived from chi square test.

There were significant differences in somatization (P = 0.002), obsessive-compulsive (P = 0.031), depression (P = 0.007), anxiety (P = 0.042), and hostility (P = 0.002) between two groups, but no significant differences were observed for other subscales. The results showed that there were also significant differences between two groups concerning the GSI (P = 0.045) and the PSDI (P < 0.001). The detailed results are shown in Table 1. However, the influence of demographic factors on psychological health status was examined and the results are shown in Table 2. In most instances being old and being in exposed group were significant factors for lower psychological health status.

**Table 2:** The relationships between the scores of SCL-90-R and demographic parameters and GSI scores

		**Age (years)**	**BMI (kg/m**^**2**^**)**	**Marital**	**Education**	**Occupation**	**Exposure**
**Somatization**	Beta	−0.002	0.008	0.248	0.050	0.065	0.313
	P-value	0.587	0.416	0.141	0.558	0.458	**0.002**
**Obsessive-compulsive**	Beta	−0.003	0.001	0.134	0.060	0.086	0.225
	P-value	0.493	0.926	0. 411	0.469	0.316	**0.019**
**Interpersonal sensitivity**	Beta	−0.012	−0.002	0.128	0.058	0.108	0.125
	P-value	**0.004**	0.857	0.397	0.450	0.173	0.161
**Depression**	Beta	−0.009	0.003	0.170	0.113	−0.063	0.304
	P-value	**0.046**	0.794	0.295	0.169	0.456	**0.002**
**Anxiety**	Beta	−0.012	0.006	0.245	0.096	0.112	0.194
	P-value	**0.005**	0.525	0.128	0.238	0.182	**0.040**
**Hostility**	Beta	−0.009	0.007	0.086	0.102	0.050	0.223
	P-value	**0.049**	0.491	0.598	0.220	0.556	**0.020**
**Phobic anxiety**	Beta	−0.010	−0.002	−0.055	−0.015	0.158	−0.020
	P-value	**0.016**	0.843	0.733	0.856	0.061	0.830
**Paranoid ideation**	Beta	−0.014	0.011	−0.064	0.086	0.338	−0.002
	P-value	**<0.001**	0.212	0.665	0.251	**<0.001**	0.978
**Psychoticism**	Beta	−0.011	−0.003	−0.086	0.036	0.101	0.069
	P-value	**0.004**	0.696	0.538	0.608	0.164	0.400
**GSI**	Beta	−0.009	0.003	0.096	0.065	0.090	0.169
	P-value	**0.021**	0.730	0.491	0.354	0.219	**0.039**
**PST**	Beta	−0.335	0.132	2.024	1.359	2.815	2.669
	P-value	**0.002**	0.602	0.628	0.520	0.197	0.276
**PSDI**	Beta	−0.002	0.001	0.107	0.068	−0.018	0.233
	P-value	0.417	0.996	0.272	0.167	0.720	**<0.001**

Beta: Regression coefficient (Married = 1) (High school and upper = 1) (Unemployed = 1) (Exposed = 1).

The results showed that a larger proportion of cases had psychological symptoms than controls (92.7% in exposed vs. 84.5% in control group; P = 0.006) (Table 3). In addition when we analyzed the data based on severity of symptoms, the results indicated that there were no significant score differences between hospitalized and non-hospitalized patients (Table 4).

**Table 3:** Psychological disorders according to the GSI index cut-off values in exposed and controls

	**Control (n = 128)**	**Exposed (n = 397)**	
	**No. (%)**	**No. (%)**	P*
**Psychological status**			0.006
**Normal (global severity index < 0.4)**	20 (15.5)	27 (7.3)	
**Probable case (global severity index ≥ 0.4)**	108 (84.5)	340 (92.7)	

* Comparison with control group derived from chi square test.

**Table 4:** Severity of the mental health disturbances among the study participants by Severity of the exposure

	**Control (n = 128)**	**Exposed (n = 367)**				
		**Non-hospitalized (n = 192)**		**Hospitalized (n = 162)**		
	**Mean (SD)**	**Mean (SD)**	P*	**Mean (SD)**	P*	P**
**Somatization**	1.50(0.88)	1.78(0.83)	0.014	1.78(0.82)	0.016	0.998
**Obsessive-compulsive**	1.37(0.85)	1.54(0.81)	0.155	1.56(0.77)	0.109	0.963
**Interpersonal sensitivity**	1.08(0.76)	1.19(0.75)	0.428	1.15(0.75)	0.707	0.891
**Depression**	1.20(0.75)	1.40(0.80)	0.073	1.45(0.82)	0.024	0.840
**Anxiety**	1.24(0.81)	1.41(0.78)	0.184	1.42(0.81)	0.146	0.978
**Hostility**	1.10(0.84)	1.37(0.79)	0.013	1.36(0.78)	0.019	0.999
**Phobic anxiety**	0.76(0.72)	0. 75(0.81)	0.999	0.71(0.82)	0.883	0.883
**Paranoid ideation**	1.20(0.80)	1.31(0.75)	0.431	1.20(0.74)	0.998	0.339
**Psychoticism**	0.78(0.66)	0.82(0.69)	0.891	0.84(0.70)	0.795	0.972
**Global severity index (GSI)**	1.16(0.70)	1.31(0.68)	0.160	1.31(0.68)	0.188	1.000
**Positive symptom total (PST)**	51.10(23.25)	53.05(19.75)	0.695	52.73(19.93)	0.788	0.989
**Positive symptom distress index (PSDI)**	1.94(0.46)	2.14(0.50)	0.001	2.15(0.46)	0.001	0.986

* Comparison with control group derived from t-test.

** Comparison between hospitalized and non-hospitalized group derived from t-test.

## Discussion

This study aimed to assess psychological distress among people who exposed to mustard gas in Sardash, Iran. The findings showed that the exposed civilians had a higher degree of psychological symptoms as compared to the controls. The global severity index (GSI) in the exposed group was significantly higher than the control group; mean values (SD) were 1.31 (0.68), 1.16 (0.70), respectively (P = 0.04). No surprisingly a recent publication from Iran also revealed that the mean GSI in survivors of chemical warfare with ophthalmologic complications was higher than standardized cut-off point for Iranian community [21]. Similarly, a Danish study among subjects who had been deployed in the Persian Gulf reported that the mean values of the GSI in Gulf War veterans were higher than controls; the mean values (SD) were 0.20 (0.26) and 0.12 (0.17), respectively (P < 0.001) [22]. It has been suggested that the increased psychological distress in Danish veterans was due to mentally distressing environment and not the neurotoxic exposure. However, lower scores in the Danish study compared with the present study might be due to several reasons. For instance, perhaps the Danish authorities implemented some effective clinical and social interventions for the Golf War veterans while we did not. Other reasons might include the fact that the Danish veterans were healthier than the Iranian civilians. There is evidence that of 34000 Iranians examined 13–20 years after exposure to SM, it was found that a considerable percentage are suffering from long term complications from exposure involving the lungs (42.5%), eyes (39%), and skin (24.5%) [6].

The results of this study showed that there were significant differences in hostility, anxiety, obsessive-compulsive, somatization and depression among the exposed and the control groups. The Danish study showed that, six dimensions on the SCL-90 including obsessive-compulsive, depression, interpersonal sensitivity, anxiety and hostility were associated with being a Gulf War veteran [22]. Interestingly both studies (the Danish and our study) did not find any significant differences in phobic, paranoid, and psychoticism among the cases and the controls. Perhaps these findings suggest that such psychological disorders are not related to neurotoxi exposure.

The development of psychiatric disorders among chemical victims is often due to severe sequelae. Studies have shown that there is strong association between physical illness with subsequent disability and psychiatric disorders in chemical warfare survivors [23-25]. Thus, one might argue that the higher rate of mental health problems in chemical warfare patients might be due to the higher rate of morbidities among this community [26]. There is evidence that exposure to mustard gas can cause both early toxic and long-term adverse health effects [27]. In addition it seems that exposure to war; adverse physical health consequences and fear of the future represent an additive effect for involved and persistent mental health [10]. However, the Sardasht-Iran Cohort Study was designed to compare those who were afflicted by sulfur mustard exposure to those who were not. Intensive conventional warfare took place in both Sardasht and Rabat, but the Sardasht cohort was comprised of victims, while the control group from Rabat was not necessarily victims (i.e., physically wounded by the conflict). Therefore, chemical burns produce more of the persistent psychological symptoms than the bullet wounds of conventional warfare.

The impact of demographic factors (other than age and being in the exposed group) on psychological health status was very little (see Table 2). Such observation indicates that the most important factor for developing psychological problems is exposure to sulfur mustard gas. Every effort to destroy such weapons seems very relevant to public’s health worldwide.

Finally, while logically the more severe exposure seems to be accompanied with more psychological problems, our study did not show that the severity of exposure affects the severity of psychological disturbances. It seems that the other confounding factors involved in this regard and needs further investigations.

### Limitations

This study had some limitations. Firstly, as noted, because of similar religion, culture, language and nutritional habits, Rabat civilians were considered as control group to compare to Sardasht exposed individuals. Since Rabat civilians themselves probably suffered from this incident and war as a big trauma indirectly so the results of this study cannot be considered as a comparison between sulfur mustard exposed and a normal population. Secondly, this study did not differentiate between the psychological symptoms induced by sulfur mustard itself and the symptoms induced by the horror and the fears in the city from heavy explosions. Thus the results should be interpreted with some cautions.

## Conclusion

This study found that civilians exposed to sulfur mustard gas were suffering from a number of psychological symptoms even 20 years after exposure. It seems that providing mental health services and more resources allocation for this community is warranted in addition to efforts to manage physical symptoms of chemical agent exposure.

## Competing interests

The authors declare that they have no competing interests.

## Authors’ contributions

RR participated in study design, questionnaire preparation and manuscript writing; PR participated in manuscript preparation; ZGH collected data and participated in manuscript preparation; AM contributed to the analysis, critically evaluated the paper, and provided the final draft; MRS participated in data collection; MMN performed the statistical analysis and interpreted data; MM and AT drafted the manuscript; TGH designed and coordinated the study. All authors read and approved the final manuscript.
